# PDGFRβ^+^/c-kit^+^ pulp cells are odontoblastic progenitors capable of producing dentin-like structure in vitro and in vivo

**DOI:** 10.1186/s12903-016-0307-8

**Published:** 2016-10-28

**Authors:** Shiwei Cai, Wenjian Zhang, Wei Chen

**Affiliations:** 1 Department of Endodontics, University of Texas School of Dentistry at Houston, 7500 Cambridge Street, Suite 5366, Houston, TX 77054 USA; 2 Department of Diagnostic and Biomedical Sciences, University of Texas School of Dentistry at Houston, 7500 Cambridge Street, Suite 5366, Houston, TX 77054 USA

**Keywords:** c-kit, Odontoblasts, Platelet-derived growth factor receptor, Pulp regeneration, Stem cell

## Abstract

**Background:**

Successful pulp regeneration depends on identification of pulp stem cells capable of differentiation under odontoblastic lineage and producing pulp-dentinal like structure. Recent studies demonstrate that platelet-derived growth factor (PDGF) plays an important role in damage repair and tissue regeneration. The aim of this study was to identify a subpopulation of dental pulp cells responsive to PDGF and with dentin regeneration potential.

**Methods:**

Pulp tissues were isolated from 12 freshly extracted human impacted third molars. Pulp cells were sorted by their expression of PDGFRβ and stem cell marker genes via flow cytometry. For the selected cells, proliferation was analyzed by a colorimetric cell proliferation assay, differentiation was assessed by real time PCR detection the expression of odontoblast marker genes, and mineralization was evaluated by Alizarin Red S staining. GFP marked PDGFRβ^+^/c-kit^+^ pulp cells were transplanted into emptied root canals of nude rat lower left incisors. Pulp-dentinal regeneration was examined by immunohistochemistry.

**Results:**

PDGFRβ^+^/c-kit^+^ pulp cells proliferated significantly faster than whole pulp cells. In mineralization media, PDGFRβ^+^/c-kit^+^ pulp cells were able to develop under odontoblastic linage as demonstrated by a progressively increased expression of DMP1, DSPP, and osteocalcin. BMP2 seemed to enhance whereas PDGF-BB seemed to inhibit odontoblastic differentiation and mineralization of PDGFRβ^+^/c-kit^+^ pulp cells. In vivo root canal transplantation study revealed globular dentin and pulp-like tissue formation by PDGFRβ^+^/c-kit^+^ cells.

**Conclusions:**

PDGFRβ^+^/c-kit^+^ pulp cells appear to have pulp stem cell potential capable of producing dentinal like structure in vitro and in vivo.

**Electronic supplementary material:**

The online version of this article (doi:10.1186/s12903-016-0307-8) contains supplementary material, which is available to authorized users.

## Background

One of the ultimate goals of endodontic therapy is to retain natural dentition via predictably regenerating vital dental pulp which is lost as a consequence of pulp diseases or trauma [1]. The cellular and molecular mechanisms regulating the formation of pulp dentinal tissues during development as well as during pulp regeneration remain to be fully elucidated.

Dental pulp is a highly vascularized and metabolically active tissue with a large capacity for regeneration [2]. In vitro study finds that pulp tissue can be used to isolate post-natal stem cells, which presumably locate at perivascular area [3]. Pulpal injury stimulates the proliferation and differentiation of the progenitor/stem cells at this area with subsequent reparative dentin formation [4].

Dental pulpal stem cells (DPSCs) isolated by affinity purification using an antibody against STRO-1, an early marker of mesenchymal stem cells, are capable of generating dentin-like structures lined with human odontoblast-like cells surrounded by a pulp-like interstitial tissue [3, 5]. Majority of these DPSCs demonstrate a phenotype consistent with pericytes, and are found to be positive for α-smooth muscle actin, CD146, as well as a pericyte marker, 3G5 [3]. These findings indicate a possible common ontogeny between dental pulp tissue and pericytes, since both are believed to originate from migratory neural crest cells during embryogenesis [6].

DPSCs have been found to demonstrate many stem cell properties, such as clonogenic efficiency, self-renewal capability, high mitotic activity, and multi-lineage differentiation capacity [7]. Depending on the culture conditions, DPSCs are able to differentiate into osteoblasts, endotheliocytes, adipocytes, neural-like cells, as well as odontoblasts [5, 7–10]. Pag A fraction of dental pulp cells have been shown to express stem cell marker genes, including STRO-1 and c-kit [3, 10, 11], indicating that these markers could be used to identify stem/progenitor cells from dental pulp.

Various degrees of success have been achieved for pulp-dentin regeneration in pulpectomized dog teeth with transplantation of DPSCs supplemented with different growth factors [12–14]. In light of the heterogeneity of pulp cells and the complexity of microenvironment in dental pulp, identify the subpopulation of pulp cells and/or growth factors with the highest regenerative potential would help to achieve optimal pulp-dentin regeneration.

In damaged tissues, hemorrhage is followed by blood clot formation and platelet degranulation and release of growth factors including PDGF, TGF-β, and EGF [15–18]. PDGF induces fibroblast proliferation and differentiation and the formation of granulation tissue in soft-tissue wound-healing models [19]. PDGF is induced during bone fracture repair and stimulates cell replication and enhance matrix protein synthesis [20, 21]. PDGF appears to play important roles in wound repair and tissue regeneration.

PDGF is a disulfide-linked dimer of two related polypeptide chains, A and B, which are assembled as heterodimers (PDGF AB) or homodimers (PDGF AA and PDGF BB) [22]. PDGF exerts its biological activity by binding to alpha (−α) or beta (−β) PDGFRs resulting in their dimerization and the subsequent activation of intrinsic receptor tyrosine kinases required to initiate cytoplasmic signal transduction pathways. Activation of these pathways is necessary to stimulate the migration, proliferation, and differentiation of PDGF-responsive cells [23]. PDGF receptors have been found to be expressed by a variety of cells, including fibroblasts, smooth muscle cells, and dental pulp cells [24–26]. The α receptor binds both PDGF A and B chains, whereas the β receptor binds only PDGF B [27]. Studies have indicated that PDGF B subunit is more potent than the A unit, and PDGFRβ occupancy permits optimal PDGF-mediated biological effects [27–30].

Dental pulp cells exhibit increased proliferative activity in the presence of PDGF-BB [24]. PDGF BB/PDGFRβ pathway appears to play an important role during terminal odontoblast differentiation [30]. It is speculated that a subpopulation of dental pulp cells positive for PDGFRβ and stem cell marker expressions would be ideal candidate for pulp stem/progenitor cells with dentin regeneration potential. The aim of the present study is to isolate dental pulp cells expressing PDGFRβ and stem cell marker by fluorescence activated cell sorting (FACS), and evaluate their responses to different growth factors and ability to produce dentin/pulp like structure in vitro and in vivo. The knowledge gained from this study is expected to help optimize regenerative endodontics, a promising alternative to root canal or implant restoration in treating pulpal and periapical diseases, and to help achieve the ultimate goal of endodontic treatment in retaining natural dentition and improving overall oral health-related quality of life.

## Methods

### Human dental pulp samples

Pulp tissue from freshly extracted normal human impacted third molars (12 subjects, 18–25 years of age) was gently separated from the crown and root, and then digested in 3 mg/ml collagenase type I and 4 mg/ml dispase for one hour at 37 °C. Single-cell suspension was obtained by passing the cells through a 70-μm strainer several times. The cells were plated at 1 × 10^5^/well into six-well gelatin coated plates in undifferentiation media, which contained 80 % Dulbecco’s Modified Eagle's medium (DMEM) and 20 % knockout serum replacement (KSR) supplemented with 1 mM MEM nonessential amino acids, 0.1 mM β-mercaptoethanol, 2 ng/ml FGF, 2 ng/ml IGF, 1 mM L-glutamine, 100 units/ml penicillin, and 100 μg/ml streptomycin. The cells were cultured at 37 °C in 5 % CO_2_ to maintain the undifferentiated status. Tooth extraction was performed with the understanding and written consent of each subject. The study was approved by the Institutional Review Board (IRB) of the University of Texas Health Sciences Center at Houston.

### Flow cytometry

**Fig. 1:**
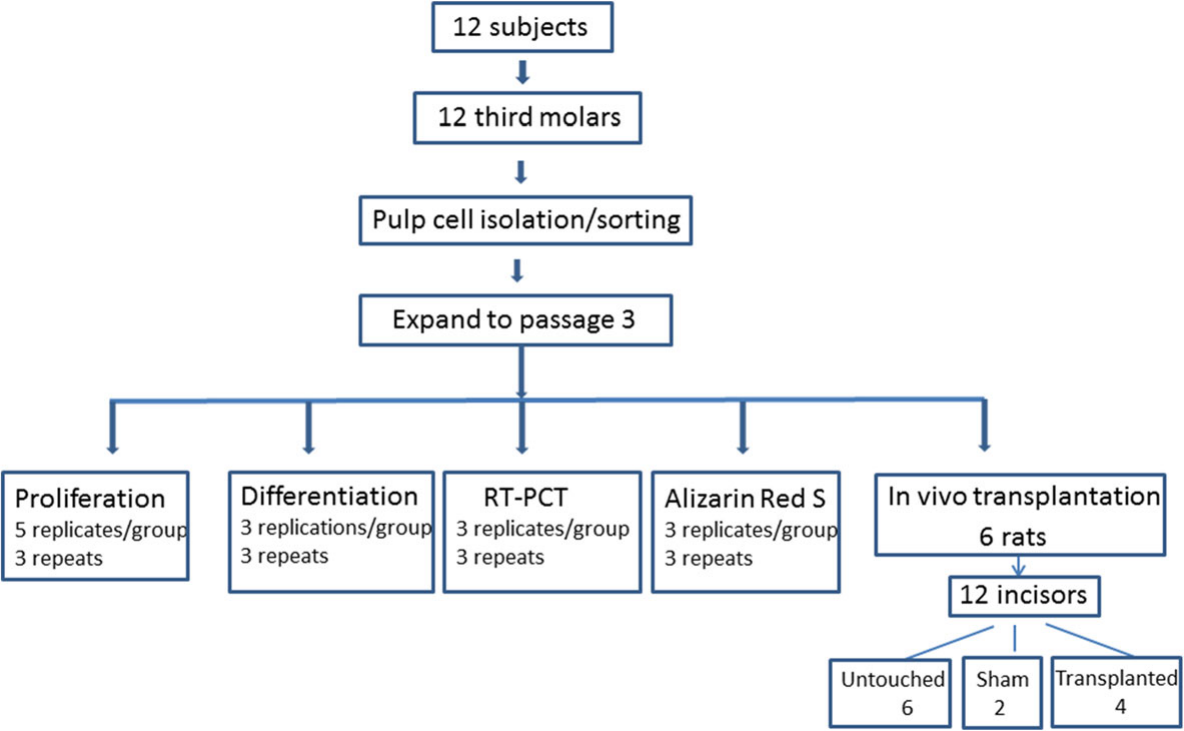
Flow chart for experimental setup

Dental pulp cells were labeled with and sorted by the reactivity to anti-PDGFRβ antibody. The PDGFRβ^+^ dental pulp cells were further characterized by their expression of stem cell and other surface markers including CD34 and c-kit (markers of hematopoietic stem cells), STRO-1 (mesenchymal stem cell marker), vimentin (mesenchymal cell marker), NG_2_ (mesenchymal cell marker), and CXCR4^+^ (chemokine receptor). The sources of these antibodies were: phycoerythrin (PE)-conjugated anti-PDGFRβ (BD Pharmingen, Franklin Lakes, NJ, USA), APC conjugated anti-CD34 (BD Pharmingen, Franklin Lakes, NJ, USA), PE-Cy5 conjugated anti-c-kit (BD Pharmingen, Franklin Lakes, NJ, USA), fluorescerin isothiocyanate (FITC)-conjugated anti-STRO-1 (Santa Cruz Biotechnology, Inc., Santa Cruz, CA, USA), FITC-conjugated anti-vimentin (Santa Cruz Biotechnology, Inc., Santa Cruz, CA, USA), FITC-conjugated anit-NG_2_ (Santa Cruz Biotechnology, Inc., Santa Cruz, CA, USA), and Allophycocyanin (APC)-conjugated anti-CXCR4^+^ (BD Pharmingen, Franklin Lakes, NJ, USA). Dental pulp cells were labelled with the antibodies for 30 min on ice followed by fluorescence sorting performed at 4 °C with a BD FACS Vantange Sorting Flow Cytometer using CELLQuest software (BD Biosciences, San Jose, CA, USA). Isotype IgG was used as control to assess background fluorescence. Isolated cells were expanded to passage 3 to ensure adequate quantity for further experiments. Figure 1 shows the flow chart for experimental setup.

### Cell proliferation assays

Cell proliferation was measured by a colorimetric assay using CellTiter 96 Aqueous One Solution (Promega, Madison, WI, USA) according to the manufacturer’s instruction. Briefly, whole pulp cells, PDGFRβ^+^ pulp cells, PDGFRβ^+^/c-kit^+^ pulp cells, and PDGFRβ^−^ cells were seeded at 2 × 10^3^ cells per well in 48-well tissue culture plates in 0.2 mL of undifferentiation media. After overnight incubation to allow cellular attachment, the media were replaced with fresh media. On days 1 to 6, 20 μl of CellTiter 96 Aqueous One Solution was added to each well. The plates were incubated in darkness at 37 °C for 1 h. Then 200 μl of the mixed medium solution was transferred to 96-well plate, and the absorbance was read at 490 nm with a SPECTRAmax 190 multiplate reader with SOFTmax PRO version 3.0 (Molecular Devices, Sunnyvale, CA, USA). The mean absorbance from wells containing cell-free media was used as the baseline and was deducted from the absorbencies of other cell-containing wells. The readings from five individual wells were averaged for each cell type at assayed time point. The proliferation rate was calculated as the following for each cell type: (absorbance at assayed time point/absorbance on day 1) X100.

### In vitro differentiation of pulp cells

To stimulate differentiation, PDGFRβ^+^/c-kit^+^ cells were cultured in mineralization media containing 90 % Minimum Essential Medium α (α – MEM) (Gibco/Thermo Fisher Scientific, Grand Island, NY, USA), 10 % fetal bovine serum (FBS), 1 mM L-glutamine, and supplemented with 50 μg/ml ascorbic acid, 10 mM β – glycerophosphate, and 10 nM dexamethasone. For concentration study, the cells were cultured in the presence and absence of 1, 10, 100, and 1000 ng/ml BMP2 or PDGF-BB for two days. The mRNA was extracted, and the expressions of odontoblast differentiation markers were detected by real-time PCR.

For the time-course study, PDGFRβ^+^/c-kit^+^ cells were cultured in mineralization media with the presence or absence of 10 ng/ml BMP2 or PDGF-BB for 2 weeks. The media were changed every other day. On days 3, 7, and 14, mRNA was extracted and real-time PCR was run to detect the expressions of odontoblast differentiation markers.

### RNA isolation and real time PCR

Total RNA was isolated from PDGFRβ^+^/c-kit^+^ cell cultures by a Qiagen Rneasy Mini Kit (Qiagen Inc, Valencia, CA, USA) according to the manufacturer’s instructions and purified via DNAse treatment. The extracted RNA had a A260/A280 ratio above 2.0. Reverse transcriptase treatment of RNA was performed at 42 °C for 80 min using 3 μg of RNA with oligo primers (Invitrogen, Carlsbad, CA, USA) and BD Sprint Powerscript (BD Biosciences, San Jose, CA, USA). The 10 μl PCR reaction mixture consisted of 4 μl cDNA dilution (40 ng cDNA), 1 μl primers (5 μM) and 5 μl 2X iQ SYBR Green Supermix (Bio-Rad Laboratories, Hercules, CA, USA). Each sample was amplificated for 40 cycles of 95 °C/10 s, 55 °C/15 s, and 72 °C/15 s with a Bio-Rad Real-Time PCR Detection System. Melting curve analysis was performed on the products to ensure specificity of the reaction. After calibrator normalization and amplification efficiency correction, PCR products were quantified by comparing amplification of the gene of interest to that of glyceraldehyde 3-phosphate dehydrogenase (GAPDH) as a reference gene, using 2 ^–ΔΔCt^ method with the Relative Quantification Software version 1.0 (Bio-Rad Laboratories, Hercules, CA, USA). The genes detected were dentin matrix protein 1 (DMP1), dentin sialophosphoprotein (DSPP), osteocalcin (OCN), and alkaline phosphatase (ALP). The criteria for choosing primers included: 18–24 bases in length, GC content 50-60 %, avoid repeats of Gs or Cs longer than 3 bases, amplified region 60–150 bp, melting temperature 50-65 °C, avoid primer-dimer formation and secondary structure in the target sequence, and verifying specificity by blasting the primers’ sequences. The primers were: DMP1, 5’- CAGGAGCACAGGAAAAGGAG and 5’- CTGGTGGTATCTTCCCCCAGGAG; DSPP, 5’- AGAAGGACCTGGCCAAAAAT and 5’- TCTCCTCGGCTACTGCTGTT; OCN, 5’- CATGAGAGCCCTCACA, and 5’- AGAGCGACACCCTAGAC; ALP, 5’- ACGTGGCTAAGAATGTCATC, and 5’- CTGGTAGGCGATGTCCTTA; and GAPDH, 5’- CGGAGTCAACGGATTTGGTCGTAT, and 5’- AGCCTTCTCCATGGTGGTGAAGAC.

### Alizarin Red S staining (ARS)

PDGFRβ^+^/c-kit^+^ pulp cells were cultured in undifferentiation media or mineralization media with the presence or absence of 1, 10, 100, and 1000 ng/ml BMP2 or PDGF-BB. The media were changed every other day, and mineral formation of the cells was assessed with ARS on day 14. Briefly, the media were removed, and the cells were rinsed with distilled water twice and fixed with 50 % ethanol at 4 °C for 45 min. Following removal of the ethanol, the cells were rinsed with deionized water twice and stained with 1 % Alizarin Red S and 0.1 % ammonium hydroxide for 30 min at room temperature. The Alizarin Red S solution was then removed, and the cells were rinsed with water three times. The stained mineral nodules within each well were documented by photomicrography with Nikon E400 microscope (Nikon, Melville, NY, USA) at 100-200X magnification. To quantify the Alizarin Red S staining, the cells were rinsed with deionized water, washed in PBS while rocking for 15 min at room temperature, then destained for 30 min with 10 % (w/v) acetic acid and 20 % (v/v) methanol extraction solution. The absorbance of extracted Alizarin Red S solution was determined at 490 nm with a microplate spectrophotometer (Bio-Rad Laboratories, Hercules, CA, USA). The concentration of Alizarin Red S in each sample was quantified by comparing the absorbance of extraction solution with those from Alizarin Red S standards.

### Plasmid constructs and transfection

The PDGFRβ clone was purchased from ATCC (Manassas, VA, USA). The cDNA of the PDGFRβ was isolated with Qiagen miniprep kit (Qiagen, Valencia, CA, USA). The cDNA of the PDGFRβ open reading frame was amplified, digested, and inserted into the pcDNA3.1/NT-GFP-ToPo plasmid vector (Invitrogen, Carlsbad, CA, USA). The recombinant PDGFRβ plasmid was transfected into PDGFRβ^+^/c-kit^+^ pulp cells using LipofectAMINE reagent and Opti-MEM (Invitrogen, Carlsbad, CA, USA) according to the manufacturer’s instructions. Forty-eight hours after transfection, cells were harvested with 0.25 % trypsin/EDTA, resuspended in DMEM with 10 % FBS, washed with PBS, and filtered through a 35 μm nylon screen. The cells were then cultured in DMEM with 10 % FBS and 400 μg/ml G418 (Sigma-Aldrich, St. Louis, MO, USA) to select the ones with successful transfection. Two weeks later, transfection efficacy was examined with a fluorescent microscope, and the transfectants (GFP marked PDGFRβ^+^/c-kit^+^ cells) were used in in vivo root canal transplantation experiments.

### In vivo root canal implantation

Six 8–10 week old NIH nude rats (NIH-Foxn 1/rnu), with body weight 150–225 g, were obtained from Charles River Laboratories (Wilmington, MA, USA). The rats were kept in isolated cages and provided with sterile housing, food, and drinking water under 12 h light/dark cycle. The animal surgery protocol was approved by the Animal Welfare Committee of the University of Texas Health Sciences Center at Houston. Animals were anesthetized with intraperitoneal injection of 135 mg/kg ketamine and 15 mg/kg xylazine. The apical region of the rat lower left incisor was surgically exposed by first making an incision through the skin and masseter using the corner of the mouth and the inferior border of the ear as landmarks, followed by removal of the external bony wall of the mandible using a high speed surgical hand piece and a #4 round long shank bur under sterile saline irrigation. The root apex and apical papillary tissues were removed by a #2 round bur and a small spoon excavator. Through the opened apex, a pre-bent #20 or #25 endodontic K file was advanced in an anterior direction into the presumed canal space, and a digital radiograph was taken to confirm the entrance of the endodontic file into the canal space. Pulpal tissue was removed with #30 or #35 barbed broaches and #20, #25, and #30 endodontic files, and the canal space was irrigated with sterile water to remove lose remnants. The procedure created an empty root canal space and adjacent bony crypt which was used as the recipient site for transplanted GFP marked PDGFRβ^+^/c-kit^+^ cells. 1 × 10^7^ cells in 50 μl Chitosan Hydrogel (Sigma-Aldrich, St. Louis, MO, USA) were implanted in the root canal space and bony crypt. The crypt was then covered with an oval piece of Collatape (Integra, Plainsboro, NJ, USA) to ensure the transplanted cells stay within the place prior to the surgical closing of the masseter and skin incision. Detailed surgical procedure is illustrated in Additional file 1: Figure S1, Additional file 2: Figure S2, Additional file 3: Figure S3, Additional file 4: Figure S4, Additional file 5: Figure S5, Additional file 6: Figure S6, Additional file 7: Figure S7, Additional file 8: Figure S8 and Additional file 9: Figure S9.

### Immunohistochemistry

Ten weeks after transplantation, mandibles were harvested, fixed in 10 % neutral buffered formalin, and decalcified in 3.4 % Kristensen’s sodium formate/15 % formic acid solution. Specimens were then dehydrated through increasing concentrations of alcohols, including 50, 70 , 95, and 100 % for 2 min each, followed by paraffin embedding. Sessions (6-micron thickness) cut through the middle of the rat incisor root canal space were mounted, deparaffinized with xylene, and rehydrated in descending concentrations of alcohols. Antigen retrieval was performed by incubation with 1 mg/ml proteinase K (Invitrogen, Carlsbad, CA, USA) for 10 min at RT. The slides were rinsed, and endogenous hydrogen peroxidase activity was blocked by incubation with PeroxyAbolish (Biocare Medical, Concord, CA, USA) for 10 min at RT. The slides were then treated with the primary antibodies overnight at 4 °C according to the manufacturers’ instructions. The primary antibodies were 1:500 rabbit anti-GFP (Abcam, Cambridge, MA, USA), 1:200 rabbit anti-PDGFRβ^+^, rabbit anti-DSP, and mouse anti-osteopontin (OPN) (Santa Cruz Biotechnology, Inc., Santa Cruz, CA, USA). Negative controls were the ones with exclusion of primary antibodies, and positive controls were the tissue samples with high level expressions for the detected proteins, such as human kidney for PDGFRβ, tooth for DSP, and bone for OPN. Sections were further incubated with 1:10,000 secondary antibodies diluted in blocking buffer. Finally, the sections were reacted with streptavidin peroxidase for 30 min at RT, stained with DAB substrate kit (Vector laboratories, Burlingame, CA, USA), and counterstained with hematoxylin (sigma-Aldrich, St. Louis, MO, USA) for 5 min.

### Data analysis

All of the experiments were performed at a minimum of three times. Data was analyzed by Student’s *t* test or ANOVA followed by a Tukey-Kramer multiple comparison test. Statistical significance was set at *p* < 0.05.

## Results

### Fractionation of pulp cells by surface markers

**Fig. 2:**
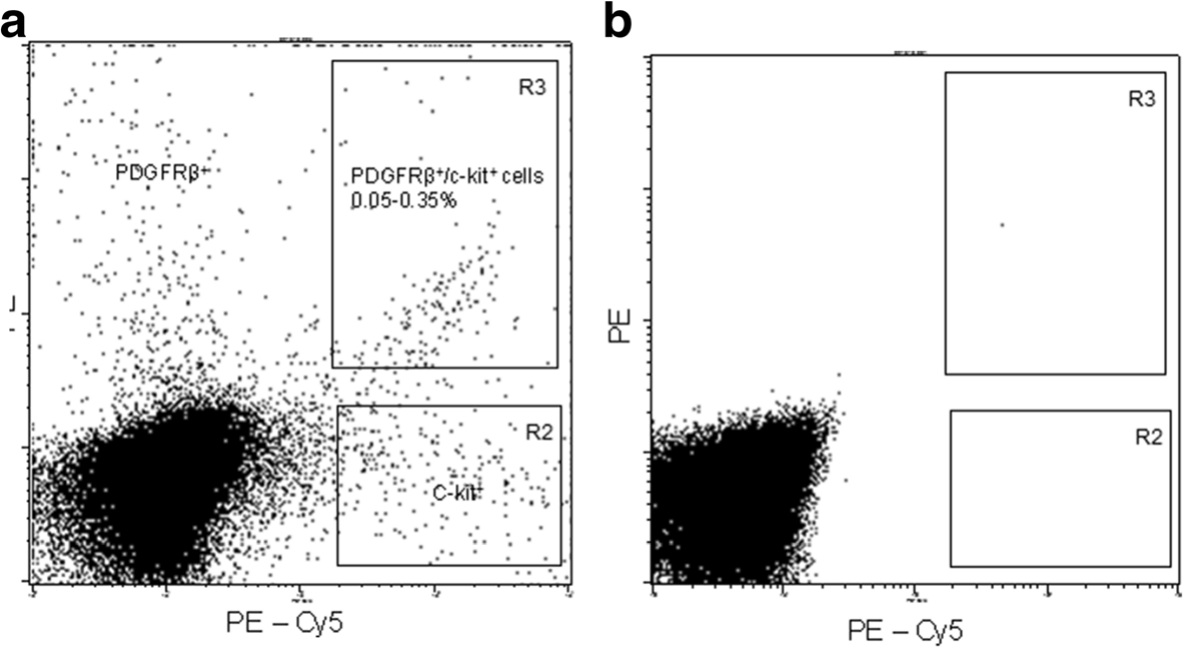
Fractionation of human dental pulp cells by fluorescence activated cell sorting (FACS). **a** Fraction of PDGFRβ^+^, c-kit^+^, and PDGFRβ^+^/c-kit^+^ cells by cell surface fluorescence labeling. **b** Isotype IgG controls

Twelve samples of adult human pulp cells were obtained from 12 individuals under 25 years of age. Cells from all 12 samples were reactive with PDGFRβ antibody, and this PDGFRβ^+^ fraction represented approximately 0.8 % of the total pulp cell population. A stem/progenitor cell population was further selected by labeling these cells with specific antigens for stem cells. Not all of the PDGFRβ^+^ cells from the 12 samples consistently reacted with STRO-1, NG_2_, CD34, vimentin, or CXCR4. However, c-kit was found to be consistently expressed by PDGFRβ^+^ cells of all 12 samples (0.15 % of the total pulp cell population) (Fig. 2). PDGFRβ^+^/c-kit^+^ cells were sorted and collected for further studies.

### PDGFRβ^+^/c-kit^+^ cells proliferated faster than whole pulp cells

**Fig. 3:**
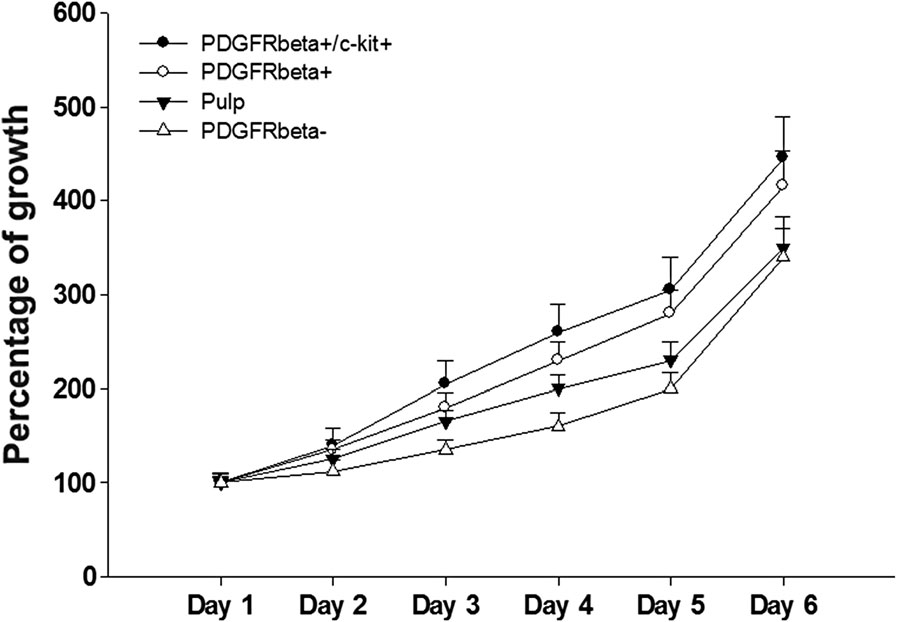
Dental pulp cell proliferation assay. In a 6-day assay period, PDGFRβ^+^/c-kit^+^ and PDGFRβ^+^ cells proliferated significantly faster than that of whole pulp cells and PDGFRβ^−^ cells from day 4 to day 6

The proliferation of whole human dental pulp cells, PDGFRβ^−^, PDGFRβ^+^, PDGFRβ^+^/c-kit^+^ cells was analyzed by a colorimetric proliferation assay through a 6-day culture period. Approximately 3 × 10^3^ cells were plated in 48-well plates instead of 96-well to prevent contact inhibition, which generated less than 90 % confluence for all the cell types at final time points. PDGFRβ^+^/c-kit^+^ and PDGFRβ^+^ cells showed significantly faster proliferation from day 4 to day 6 compared with whole pulp cells and PDGFRβ^−^ cells (*p* < 0.05). There was no significant difference of cell growth between PDGFRβ^+^/c-kit^+^ and PDGFRβ^+^ cells (Fig. 3).

### PDGFRβ^+^/c-kit^+^ cells expressed odontoblast differentiation marker genes

**Fig. 4:**
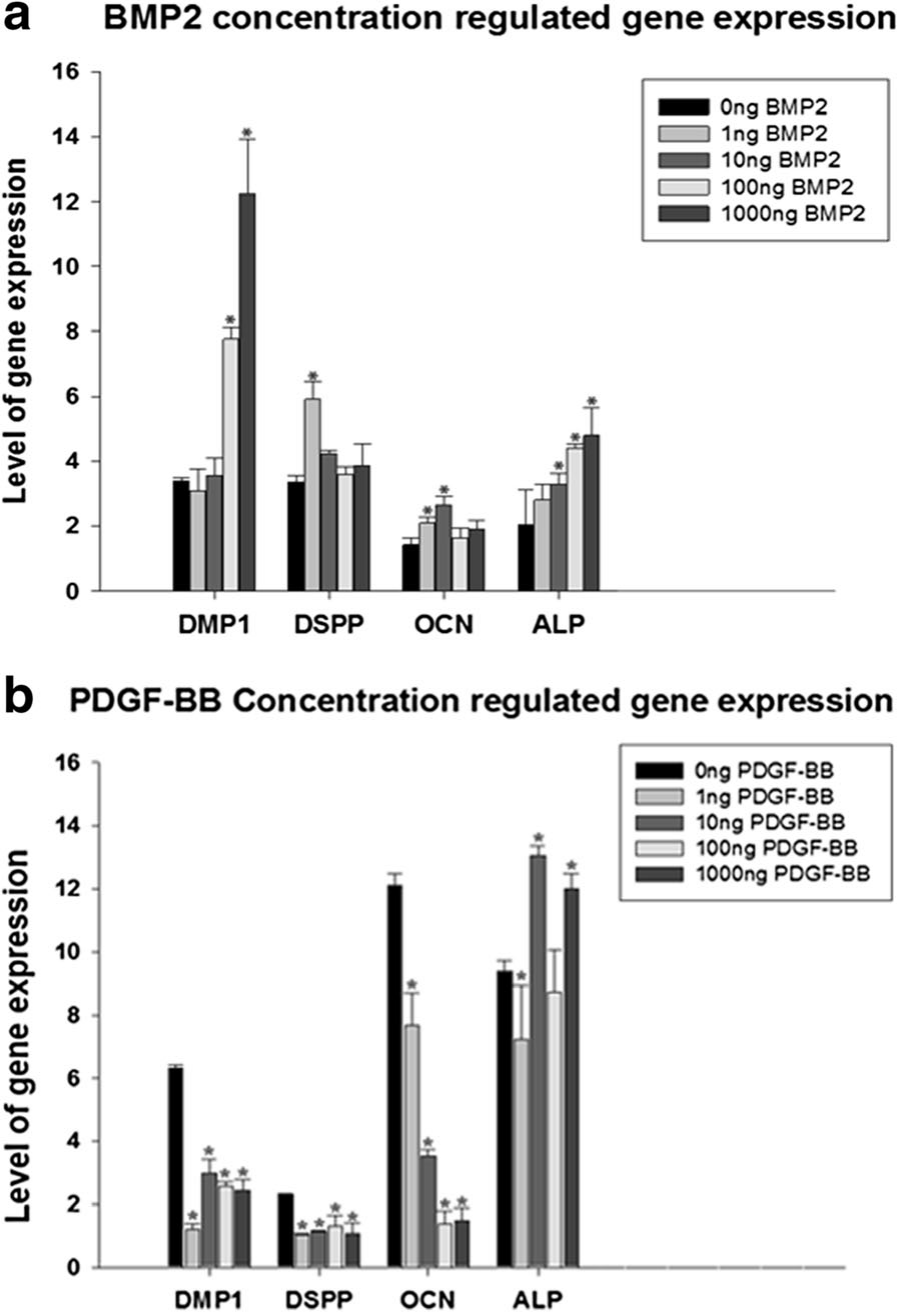
Differentiation of PDGFRβ^+^/c-kit^+^ pulp cells under various concentrations of growth factors. **a** 0–1000 ng/ml of BMP2 treatment. Expressions of DMP1, OCN, and ALP were up-regulated by BMP2 in a concentration-dependent manner. DSPP was up-regulated by 1 ng/ml BMP2. * denotes *p* < 0.05 compared with 0 ng/ml BMP2. **b** 0–1000 ng/ml of PDGF-BB treatment. Expression of OCN was down-regulated by PDGF-BB in a concentration-dependent manner. DMP1 and DSPP were inhibited in a non-concentration dependent manner. The effects on ALP were varied. * denotes *p* < 0.05 compared with 0 ng/ml PDGF-BB

For the concentration study, when PDGFRβ^+^/c-kit^+^ pulp cells were treated with 0, 1, 10, 100, and 1000 ng/ml BMP2, mRNA expressions of DMP1, OCN, and ALP were up-regulated by BMP2 in a concentration-dependent manner. DSPP was up-regulated by 1 ng/ml BMP2 (Fig. 4a).

When PDGFRβ^+^/c-kit^+^ pulp cells were treated with 0, 1, 10, 100, and 1000 ng/ml PDGF-BB, mRNA expressions of OCN was down-regulated by PDGF-BB in a concentration-dependent manner, the expressions of DMP1 and DSPP were inhibited in a non-concentration dependent manner, and the effects on ALP were varied (Fig. 4b).

**Fig. 5:**
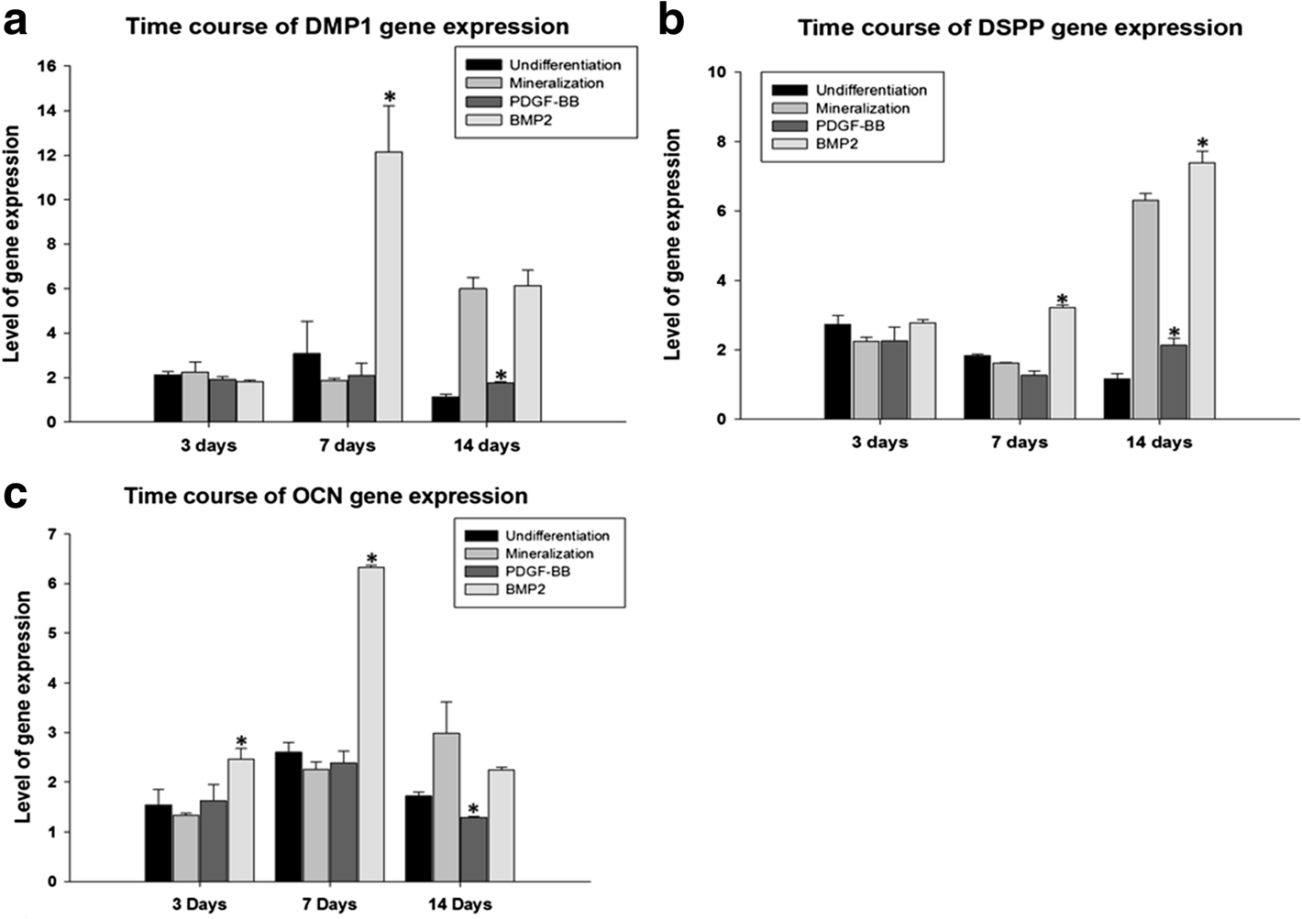
Fourteen-day differentiation study of PDGFRβ^+^/c-kit^+^ pulp cells under 10 ng/ml growth factor treatments. **a** BMP2 stimulated maximal DMP1 expressions on day 7. PDGF-BB inhibited DMP1 on day 14 when compared with mineralization media alone. **b** Compared with mineralization media alone, BMP2 significantly enhanced DSPP expressions on day 7 and 14, and PDGF-BB greatly inhibited DSPP on day 14. **c**. Compared with mineralization media alone, BMP2 significantly increased OCN expressions on day 3 and 7, and PDGF-BB greatly inhibited OCN on day 14. * denotes *p* < 0.05 compared with mineralization media alone

For the time course study, when PDGFRβ^+^/c-kit^+^ pulp cells were cultured in mineralization media alone, the expression of DMP1, DSPP, and OCN reached the highest levels on day 14. BMP2 stimulated maximal DMP1 and OCN expressions on day 7, and DSPP expression increased continuously throughout the 14 day culture period. PDGF-BB showed a general inhibitory effect on the level of DMP1, DSPP, and OCN gene expressions compared with mineralization media alone in the time course study (Fig. 5a, b, and c).

### PDGFRβ^+^/c-kit^+^ cells were able to produce mineralization in vitro

**Fig. 6:**
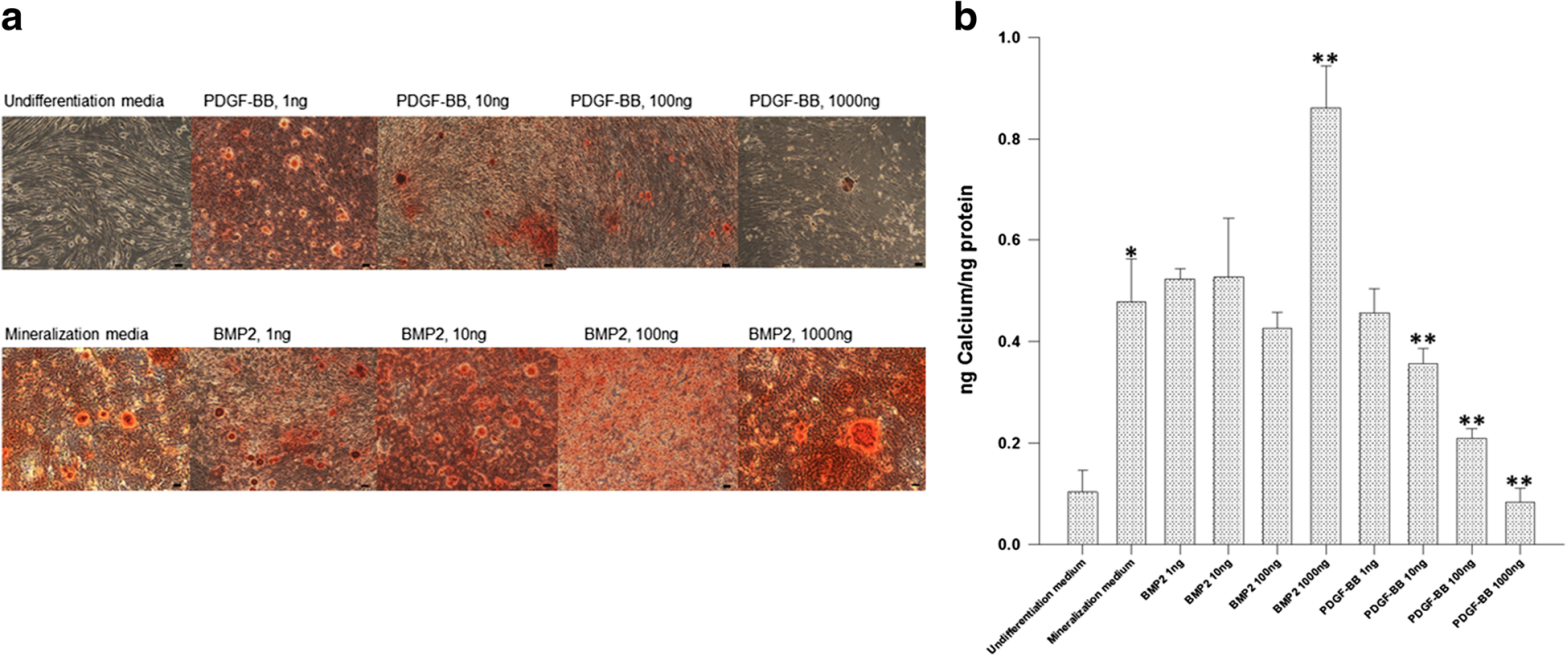
BMP2 stimulated and PDGF-BB inhibited mineralization of PDGFRβ^+^/c-kit^+^ pulp cells. **a** Alizarin Red S staining. Dark red area indicates mineralization in the culture. Scale bar = 100 μm. **b** Quantitative Alizarin Red S analysis. PDGFRβ^+^/c-kit^+^ pulp cells produced significantly more mineralization when cultured in mineralization media than that of the nondifferentiation media. 1000 ng/ml BMP2 stimulated significantly more mineralization by PDGFRβ^+^/c-kit^+^ cells than in mineralization media alone. PDGF-BB suppressed mineralization in a concentration dependent manner when compared with mineralization media alone. * denotes *p* < 0.05 compared with undifferentiation media. ** denote *p* < 0.05 compared with mineralization media

PDGFRβ^+^/c-kit^+^ pulp cells produced significantly more mineral nodules when cultured in mineralization media than that of the nondifferentiation media. Among the studied concentrations (1–1000 ng/ml), 1000 ng/ml BMP2 stimulated significantly more mineralization by PDGFRβ^+^/c-kit^+^ cells than in mineralization media alone. Interestingly, PDGF-BB suppressed the formation of mineral nodules in a concentration dependent manner than mineralization media alone (Fig. 6a, b).

### Transplanted PDGFRβ^+^/c-kit^+^ cells produced dentin-pulp like structure in vivo

**Fig. 7:**
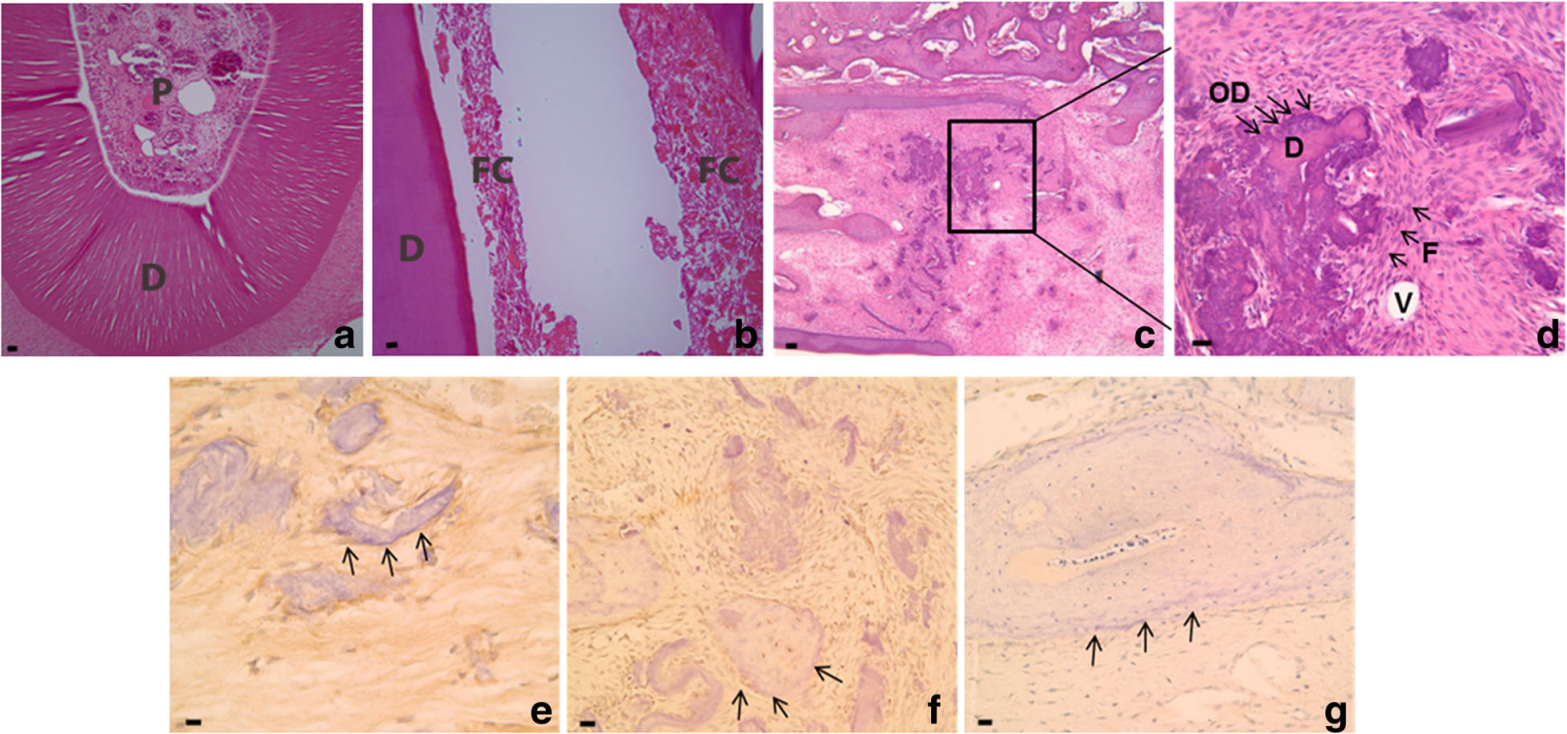
Generation of dentin-pulp like structures by PDGFRβ^+^/c-kit^+^ cells transplanted in emptied rat incisor root canal. **a** An untouched rat lower incisor as negative control. **b** A sham operation control with transplantation of hydrogel alone in rat emptied incisor root canal. Notice largely void canal space with small amount residual fibrous tissue at the periphery. **c**-**g** Transplantation of PDGFRβ^+^/c-kit^+^ cells and hydrogel into emptied rat incisor root canal. The canal was filled with globular dentin like calcification lined by odontoblast-like cells surrounded by vascular and fibrous connective tissues. **c** Low magnification **d** High magnification of the boxed area in **c**. **e** Odontoblast-like cells (arrows pointed) were positive for GFP staining. **f** Odontoblast-like cells (arrows pointed) were positive for DSP staining. **g** Odontoblast-like cells (arrows pointed) were positive for OPN staining. D, dentin; F, fibroblasts; FC, fibrous connective tissue; OD, odontoblast-like cells; P, pulp; V, blood vessel. Scale bar = 100 μm

Successful creation of recombinant PDGFRβ-GFP plasmid was confirmed by DNA sequencing. The recombinant plasmid was transfected into PDGFRβ^+^/c-kit^+^ cells, which were then transplanted into emptied rat incisor root canal. Unoperated rat lower incisors were included as negative controls (Fig. 7a). Ten weeks after transplantation, control animals that were sham-operated without transplantation of PDGFRβ^+^/c-kit^+^ pulp cells were not observed to undergo any dentinogenesis (Fig. 7b). For the animals received PDGFRβ^+^/c-kit^+^ pulp cells, some globular calcifications were developed in the emptied root canal space (Fig. 7c, d). The cells outlining the mineral surface had been shown to express moderate GFP (Fig. 7e), DSP (Fig. 7f), and weak OPN (Fig. 7g) by immunohistochemical staining. These globular dentin-like structures were surrounded by fibroblasts and vascular-like entities (Fig. 7d).

## Discussion

Our study identified 0.15 % of the total pulp cells are positive for PDGFRβ/c-kit. C-kit, a stem cell marker, has previously been shown to be expressed in dental follicle and dental papilla in vivo and in human dental pulp cells in vitro [11, 31]. PDGF-PDGFR pathway is critical for tissue repair and regeneration [19, 20]. Our study is the first one to isolate and characterize pulps cells with co-expression of c-kit and PDGFRβ, and the results suggest a promising cellular source for endodontic regeneration. The PDGFRβ^+^/c-kit^+^ pulp cells are highly proliferative, and capable of generating dentin-like structure in vitro as well as in emptied root canal space. BMP2 and PDGF-BB counteract each other and are crucial in regulating the development of PDGFRβ^+^/c-kit^+^ pulp cells into dentin-forming cells.

During differentiation phase, odontoblasts express an array of differentiation markers. DMP1 and DSPP are generally considered to be specific markers of odontoblast differentiation [32–34]. Noncollagenous proteins such as alkaline phosphatase (ALP), osteocalcin (OCN), and osteopontin (OPN) are also found in odontoblasts during dentin matrix maturation and mineralization [35, 36]. In this study, PDGFRβ^+^/c-kit^+^ cells were able to develop under odontoblastic linage when cultured in mineralization media alone, as demonstrated by a general increased expression of DMP1, DSPP, and OCN during the 14-day culture period. BMP2 stimulated the expressions of DMP1, DSPP, ALP, and OCN, whereas PDGF-BB suppressed the expressions of DMP1, DSPP, and OCN, and had a variable effect on ALP depending on concentrations. These observations suggest that BMP2 enhances but PDGF-BB inhibits differentiation of PDGFRβ^+^/c-kit^+^ cells into odontoblastic like cells. Consistently, the quantitative mineralization assay demonstrated that PDGFRβ^+^/c-kit^+^ cells were able to produce mineralized nodules in mineralization media alone, and BMP2 enhanced but PDGF-BB inhibited the mineralization process.

Majority of our findings on the effects of BMP-2 and PDGF-BB on odontoblast differentiation and mineralization are consistent with what have been reported in the literature. BMP2 can stimulate the expressions of DSPP, ALP, and OCN of dental pulp cells, and induce dentin formation in pulp cultures and pulp cap therapy [37–40]. PDGF-BB has been found to stimulate pulp cell proliferation, suppress ALP and OCN expression, and decrease calcium content and the formation of dentin-like nodules in dental pulp cultures, although DSP expression has been enhanced [24, 26, 30, 41]. The discrepancy of the effect of PDGF-BB on DSP expression in our study could be due to differences in concentrations of the growth factor, culture method, or origin of the pulp cells. Our results seem to indicate that PDGF-BB is mainly to maintain primitive status and proliferative capacity of pulp progenitor cells.

In vivo transplantation of dental pulp progenitor cells for dentinogenesis has been established previously, such as subcutaneously [5, 7] or in renal capsules [42]. The root canal space of rat incisors has not been reported as a recipient site before. As rat incisor continuously erupts, the coronal access to its root canal is restricted. In addition, rat incisor has relatively large periapical tissue mass and apex opening, which limit it being used as a recipient site for pulp stem cell transplantation. However, some features of rats make them a good model to study pulpal regeneration, such as cost effectiveness, well-established genome and proteome, and readily availability for a variety of transgenic rat models [43, 44]. Nude rats are especially suitable for transplantation study, due to its immune tolerance. To cope with continuous eruption of rat incisors, a retrograde approach was used in this study to remove the apex and periapical growing apparatus. The existing dental pulp was removed and a large empty root canal space was prepared for transplantation of human dental pulp progenitor cells. It was discovered that transplantation of PDGFRβ^+^/c-kit^+^ pulp cells into nude rat emptied incisor root canal generated globular dentin like structure with surrounding odontoblastic cells and pulp-like tissues. Immunohistochemistry revealed expression of DSP and OPN in these “dentinogenic” cells. The interface between dentin wall of rat incisor root and the transplants was filled with fibrous connective tissue and scattered vascular-like structures, which, presumably, were derive from angiogenesis of PDGFRβ^+^/c-kit^+^ cells under stimulation of local growth factors. For sham operation with transplantation of hydrogel alone, the root canal space was largely void, with small amount of fibrous connective tissue seen at the periphery, which was likely to be residual tissue left off the operation. Together, these observations validate the feasibility of the emptied root canal as alternative recipient site for dentin regeneration, and revealed the stem/progenitor cell characteristic of PDGFRβ^+^/c-kit^+^ pulp cells with regenerative potential for dentin-pulpal complex.

One limitation of the current technique was lack of fully organized dentin-pulp formation in the emptied root canal space. PDGFRβ^+^/c-kit^+^ cells appeared to provide sufficient cellular resources to generate various components of dentin-pulp complex, supplementing with optimal scaffold system and/or morphogenic factors in the local environment might facilitate a more organized de novo dentin-pulp regeneration. A variety of scaffold materials have been tested in endodontic regeneration with variable results, including injectable hydrogels, platelet-rich plasma, bioceramic, and polymer scaffolds [45–48]. Future studies using PDGFRβ^+^/c-kit^+^ cells, functionalized scaffolds, and properly titrated morphogens are needed to optimize the technique to achieve a more successful de novo regeneration of dentin-pulp complex. This study, together with other investigations in the field, strives to fulfill the ultimate goal of endodontic therapy, to retain infected or traumatized nature dentition, and avoid the discomfort and complications resulting from tooth devitalization/extraction or prosthetic restorations.

## Conclusions

In summary, the research described herein identified a subset of dental pulp cells (PDGFRβ^+^/c-kit^+^) with pulp stem cell properties such as highly proliferative and capable of producing dentin-like structures in vitro and in vivo. This knowledge will help us better understand the fundamentals of dentinogenesis and develop novel strategy for endodontic dentin/pulp regeneration.

## Additional files

Additional file 1: Figure S1. General anesthesia. (JPG 22 kb)
Additional file 2: Figure S2 Incision. (JPG 17 kb)
Additional file 3: Figure S3. Locating of apical area of incisor. (JPG 18 kb)
Additional file 4: Figure S4. Removal of apex, access of root canal through apex, and removal of pulpal tissue. (JPG 21 kb)
Additional file 5: Figure S5. Radiograph shows removal of pulp and access to canal from apex. (JPG 13 kb)
Additional file 6: Figure S6. Mixture of pulpal stem cells and gel scaffold. (JPG 39 kb)
Additional file 7: Figure S7. Implantation of cells and scaffold into emptied root canal. (JPG 18 kb)
Additional file 8: Figure S8. Suture. (JPG 22 kb)
Additional file 9: Figure S9. Collecting specimen. (JPG 29 kb)

## Abbreviations

ALP: Alkaline phosphatase
APC: Allophycocyanin
ARS: Alizarin red S staining
DMEM: Dulbecco’s modified eagle’s medium
DMP1: Dentin matrix protein 1
DPSCs: Dental pulpal stem cells
DSPP: Dentin sialophosphoprotein
FACS: Fluorescence activated cell sorting
FBS: Fetal bovine serum
FITC: Fluorescerin isothiocyanate
GAPDH: Glyceraldehyde 3-phosphate dehydrogenase
IRB: Institutional Review Board
KSR: Knockout serum replacement
OCN: Osteocalcin
OPN: Osteopontin
PDGF: Platelet-derived growth factor
PE: Phycoerythrin
α – MEM: Minimum essential medium α
