# Supplementary figures and images for: PDGFRβ+/c-kit+ pulp cells are odontoblastic progenitors capable of producing dentin-like structure in vitro and in vivo
Source: BMC Oral Health. 2016 Oct 28;16:113. doi: 10.1186/s12903-016-0307-8 (PMC5086066; doi:10.1186/s12903-016-0307-8)

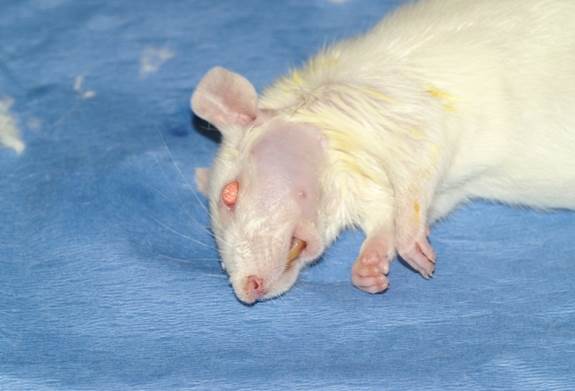

Supplement: Additional file 1: Figure S1. — General anesthesia. (JPG 22 kb) [file 12903_2016_307_MOESM1_ESM.jpg]

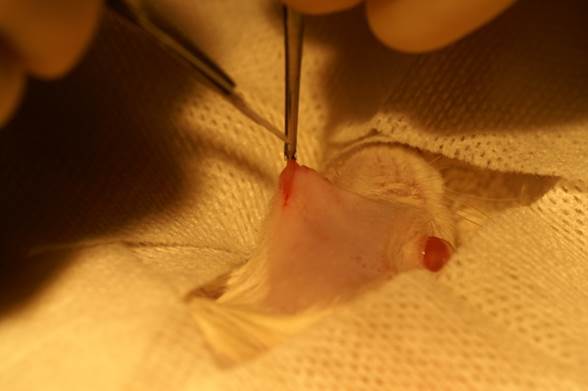

Supplement: Additional file 2: Figure S2 — Incision. (JPG 17 kb) [file 12903_2016_307_MOESM2_ESM.jpg]

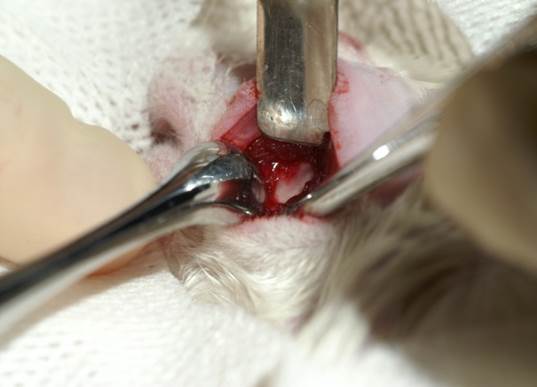

Supplement: Additional file 3: Figure S3. — Locating of apical area of incisor. (JPG 18 kb) [file 12903_2016_307_MOESM3_ESM.jpg]

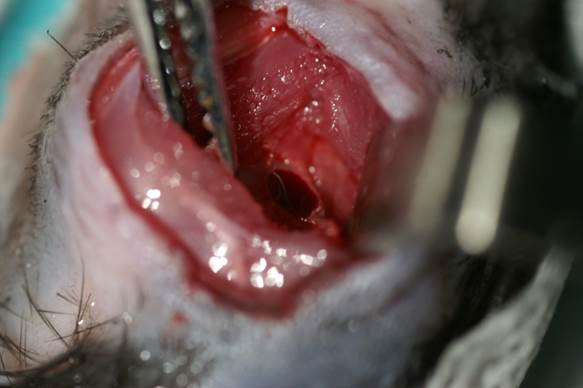

Supplement: Additional file 4: Figure S4. — Removal of apex, access of root canal through apex, and removal of pulpal tissue. (JPG 21 kb) [file 12903_2016_307_MOESM4_ESM.jpg]

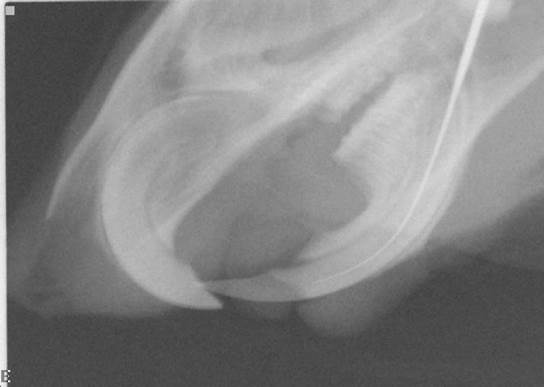

Supplement: Additional file 5: Figure S5. — Radiograph shows removal of pulp and access to canal from apex. (JPG 13 kb) [file 12903_2016_307_MOESM5_ESM.jpg]

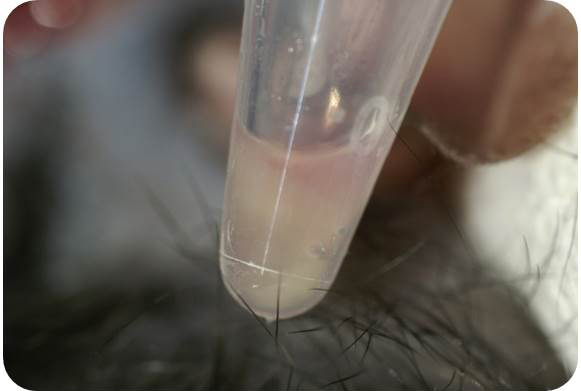

Supplement: Additional file 6: Figure S6. — Mixture of pulpal stem cells and gel scaffold. (JPG 39 kb) [file 12903_2016_307_MOESM6_ESM.jpg]

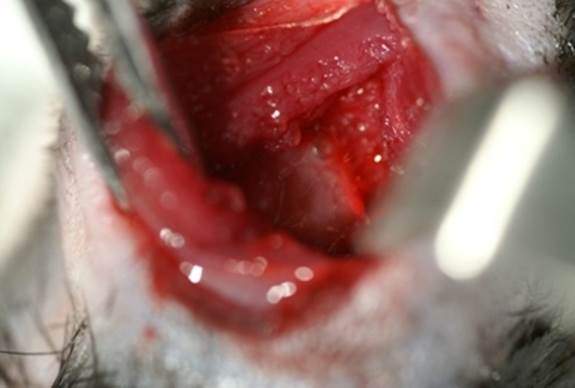

Supplement: Additional file 7: Figure S7. — Implantation of cells and scaffold into emptied root canal. (JPG 18 kb) [file 12903_2016_307_MOESM7_ESM.jpg]

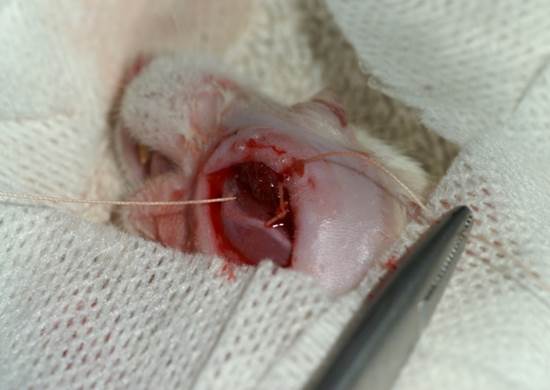

Supplement: Additional file 8: Figure S8. — Suture. (JPG 22 kb) [file 12903_2016_307_MOESM8_ESM.jpg]

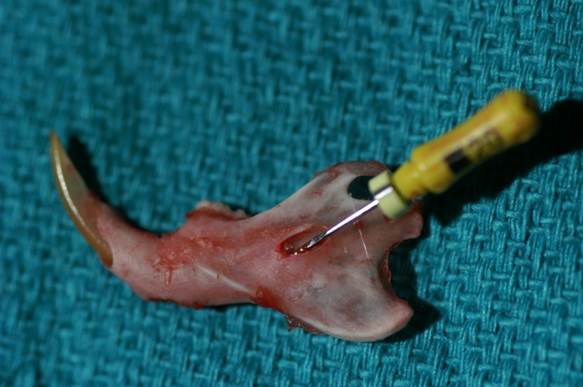

Supplement: Additional file 9: Figure S9. — Collecting specimen. (JPG 29 kb) [file 12903_2016_307_MOESM9_ESM.jpg]
